# Association between dietary vitamin B6 intake and constipation: a population-based study

**DOI:** 10.3389/fnut.2024.1483515

**Published:** 2024-11-08

**Authors:** Xuefeng Liu, Yuedong Liu, Yuping Shu, Hongwu Tao, Zewei Sheng, Yuyu Peng, Meiqi Cai, Xiaoming Zhang, Weiru Lan

**Affiliations:** ^1^Liaoning University of Traditional Chinese Medicine, Second Clinical College, Shenyang, China; ^2^Third Affiliated Hospital, Liaoning University of Traditional Chinese Medicine, Shenyang, China; ^3^Fujian Provincial People’s Hospital, Fuzhou, China

**Keywords:** vitamin B6, constipation, NHANES, dietary intake, population-based study

## Abstract

**Background:**

Numerous studies have suggested a link between dietary micronutrient intake and the onset of constipation. Nevertheless, there has not been much research done on the potential relationship between vitamin B6 and constipation. The purpose of this study was to determine whether dietary vitamin B6 consumption and chronic constipation are related among adult participants in the National Health and Nutrition Examination Survey (NHANES).

**Method:**

The study made use of information from the 2009–2010 NHANES health and nutrition survey. Respondents’ dietary information was gathered using 24-h dietary recalls. A range of statistical techniques, including as interaction tests, subgroup analyses, and curve fitting analyses, were used to examine the connection between dietary vitamin B6 intake and chronic constipation.

**Result:**

This study included 3,643 patients, with 270 (7.41%) diagnosed with persistent constipation. A fully adjusted multiple logistic regression analysis found that increasing dietary vitamin B6 consumption (OR = 0.78, 95% CI: 0.68–0.89) was linked to a lower incidence of constipation, with significance at *p* < 0.05. After accounting for numerous factors, the odds ratio and 95% confidence interval for the third tertile compared to the reference group (first tertile) were 0.85 (0.74, 0.98), with statistical significance at *p* < 0.05. Furthermore, subgroup analysis and interaction assessments revealed a substantial negative link between vitamin B6 intake and the occurrence of constipation, particularly in males and alcohol drinkers (all *p*-values were less than 0.05).

**Conclusion:**

This study found an inverse connection between vitamin B6 consumption and the prevalence of persistent constipation. More extensive prospective trials are needed to fully examine the long-term influence of vitamin B6 on persistent constipation.

## 1 Background

Constipation symptoms are experienced by 10 to 20% of persons worldwide. Traditionally, hard stools or infrequent bowel motions have been associated with the term “constipation.” This condition is heterogeneous, though, and people may experience a variety of symptoms, including hard stools, straining during defecation, a feeling of incomplete bowel evacuation, anal blockage, or the requirement for manual assistance to facilitate stool passage (1, 2).

The affected person’s social, emotional, and physical health may suffer. However, a mere 20% of people experiencing constipation consult a physician. Because of the significant incidence of this problem, about 8 million healthcare visits (3) and $230 million in costs (4) are incurred annually in the United States. Prolonged constipation has complex and multiple reasons, including intestinal neurotransmitter regulation, fluid transport, colonic motility, colonic sensory dysfunction, anorectal function, and dietary and lifestyle variables. Crucially, lifestyle choices and eating patterns have a big influence on this illness (5). Changes to these components are thought to be important, controllable causes of persistent constipation.

B vitamins are water-soluble multivitamins that are essential for anabolic and catabolic activities. The body has to replace these vitamins every day because it is unable to store them. Thiamine (B1), riboflavin (B2), niacin (B3), pantothenic acid, pyridoxine (B6), biotin, folic acid, and cobalamin (B12) are a few examples of B vitamins (6). These vitamins support several physiological processes at the cellular level and act as coenzymes in a range of enzymatic activities. Through its effects on the citric acid cycle and the electron transport chain, a shortage in B vitamins can impair the mitochondrial metabolism of amino acids, glucose, and fatty acids. This can ultimately affect vital bodily systems such as the neurological and digestive systems (7, 8). Studies indicate a definite correlation between low levels of B complex vitamins and problems including constipation, as well as decreased secretion of digestive juice and gastrointestinal motility (9). B6 is a unique kind of vitamin among the B vitamins. This water-soluble vitamin is essential for the gastrointestinal system’s digestion of proteins, lipids, and carbohydrates (10). Vitamin B6, also referred to as pyridoxine, includes pyridoxal, pyridoxamine, and pyridoxine itself. Water-soluble vitamins like vitamin B6 are mostly found in the body as phosphate esters. In 1936, vitamin B6 was given its official nomenclature. It seems to be a transparent crystal (11). Furthermore, vitamin B6 is an essential component of some coenzymes that are present in humans and are involved in a number of metabolic activities, including those related to the metabolism of amino acids. Certain foods increase the effectiveness of vitamin B6, which in turn increases its functionality (12). This improvement is facilitated by a number of nutrients, including minerals like magnesium and potassium and vitamins B complex, B1, B2, and pantothenic acid. Pregnant women’s studies suggest a possible link between low vitamin B6 levels and gastrointestinal problems, like early pregnancy vomiting (13).

There is still a great deal to learn about the possible contribution of particular vitamins to the relief of constipation, despite the fact that several research have examined the effects of different dietary elements on this illness, such as fiber consumption, certain mineral micronutrients, and fat intake. For instance, research has shown a connection between a lack of vitamin D and a higher chance of constipation (14), as well as a link between a higher risk of constipation and vitamin B6 consumption in the diet (15). However, there has not been much research done on the connection between constipation and other water-soluble vitamins, particularly vitamin B6. There is a dearth of research specifically examining how vitamin B6 affects constipation, particularly in large, population-based cohorts.

Comprehensive population studies particularly examining the relationship between vitamin B6 intake and chronic constipation are scarce, despite the fact that the body of literature already in existence discusses the connection between dietary micronutrient consumption and chronic constipation. Given this gap, the main goal of this study was to find out whether a higher dietary intake of vitamin B6 is associated with a lower incidence of chronic constipation in the general population (16).

## 2 Method

### 2.1 Survey description

**Figure 1:**
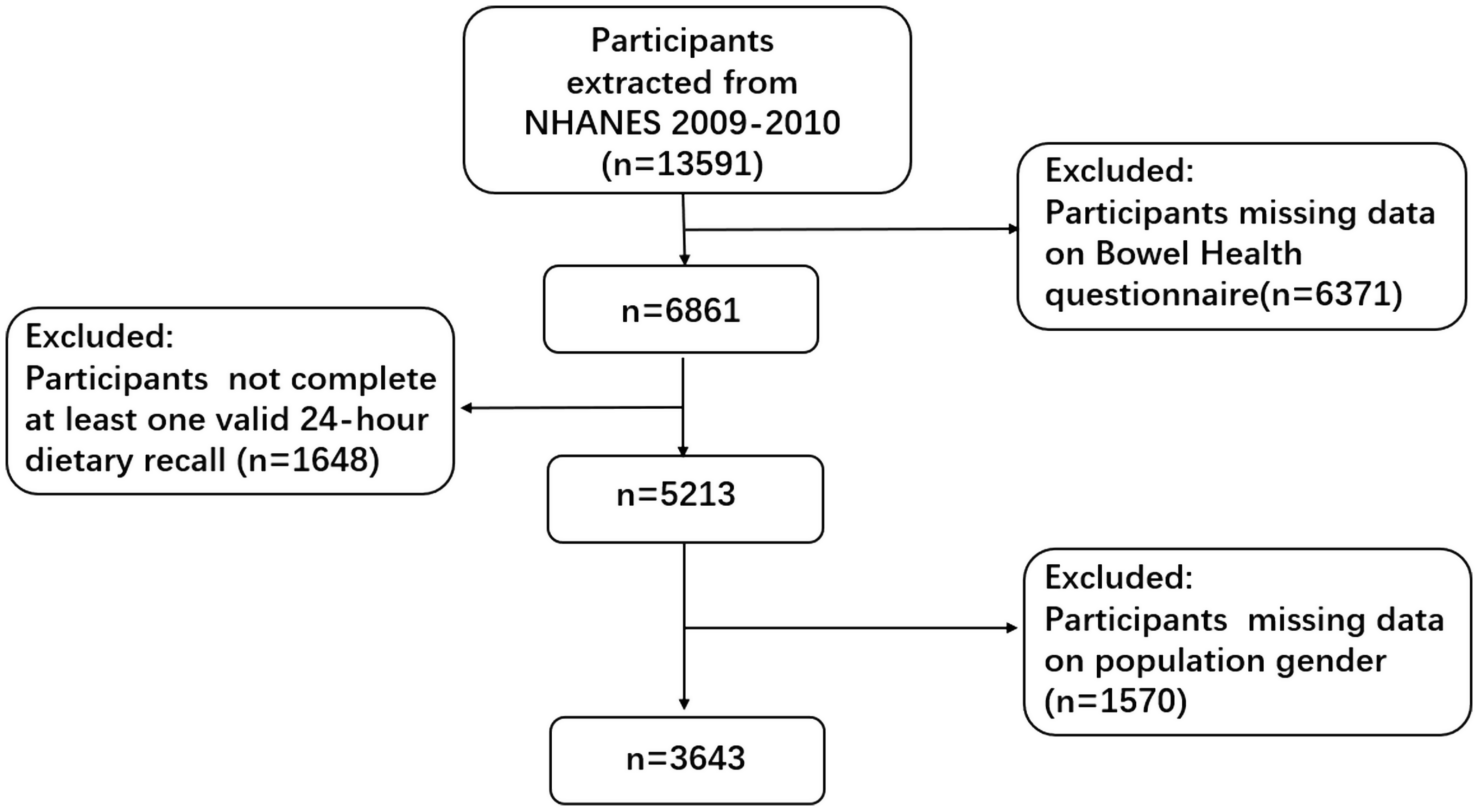
2009–2010 NHANES participant selection flowchart.

A thorough survey carried out in the US is called the NHANES program. Data from a sample that is typical of the population was gathered for this cross-sectional study design. This survey was carried out by the Centers for Disease Control’s National Center for Health Statistics (17). Using a methodology that incorporates stratified, multistage, and probability sampling techniques, the effort gathers health-related data from the broader U.S. population. NHANES’s primary goal is to gather information about the general health and eating patterns of Americans while also evaluating the nation’s adult and pediatric populations’ nutritional and health conditions. The National Center for Health Statistics’ Research Ethics Review Board approved this study, and each participant provided written consent (18). We used publicly available gut health data from the 2009–2010 NHANES period, which included 13,591 participants who were 20 years of age or older, for our research. In order to gather information about their overall stool features and frequency of bowel movements, participants were asked to complete a questionnaire. Specific exclusion criteria were established in order to guarantee the reliability and correctness of the results: people without intestinal health questionnaire data (*N* = 6,370), people without unrecorded dietary energy intake data (*N* = 1,648), and people without other covariate information (*N* = 1,570) (19, 20). In the end, 3,643 people made up the sample for the analysis. For specific information on the patient screening process, please refer to Figure 1.

### 2.2 The definition of constipation

Constipation was defined using the NHANES database, which took into account variables such stool consistency and frequency of bowel motions (21). Prior to data collection, participants documented the texture and frequency of their stools for 30 days. The Bristol Stool Form Scale (BSFS), which consists of cards with different colored pictures and explanations of seven stool kinds, was used to measure the consistency of their stools. The participants were asked to choose the number that most closely corresponded with the sort of feces that they usually saw. BSFS type 1, which is characterized by hard, nut-like clumps, or type 2, which is sausage-like but clumpy, are used to diagnose constipation. Standard classifications of stool consistency include BSFS type 3 (smooth and soft, like a sausage or snake), type 4 (smooth and soft), or type 5 (soft patches with sharp edges). The presence of either BSFS type 7, which is watery in form and lacks solid debris, or type 6, which comprises fluffy debris with rough edges and a mushy texture, indicates the existence of chronic diarrhea. There are two forms of constipation: type 1 and type 2. Stool types 3–7 are considered typical (22). Data on vitamin B6 intake was collected using a multichannel method through 24-h dietary recalls. This approach employed a respondent-driven strategy to obtain detailed information on all foods and beverages consumed by individuals within a 24-h timeframe, starting at midnight. Each participant underwent two interviews for total nutrient intake recall over 24 h. The first was conducted in person at a mobile screening site, while subsequent interviews were done over the phone within a range of 3 to 10 days. When participants completed both recalls, the average vitamin B6 intake was calculated; if not, data from the initial interview was utilized. The consumption of vitamin B6 from food sources is the primary focus of our investigation; any consumption of vitamin B6 from dietary supplements is not included.

In this study, vitamin B6 intake was categorized in intervals of three-quarters (23).

### 2.3 Covariate

This study considered a number of covariates, such as age, gender, ethnicity, body mass index (BMI), marital status, drinking habits, tobacco use, the household income-to-poverty ratio (PIR), as well as diabetes and hypertension, to reduce the impact of potential confounding variables (24, 25). Mexican Americans, other non-Hispanic groupings, non-Hispanic White, non-Hispanic Black, and other ethnicities were the classifications used to identify the various ethnic groups. Married, widowed, divorced, separated, single, cohabiting, and those who choose not to report their status were the seven groups into which marital status was segregated. The participants were split into two groups: smokers (those who presently smoke and had smoked at least 100 cigarettes in their lifetime) and never smokers (those who had never smoked or had smoked less than 100 cigarettes in their lifetime). A person was considered a drinker if, between 2009 and 2010, they drank at least 12 alcoholic beverages. A glycosylated hemoglobin test result of ≥6.5% or a self-reported physician diagnosis were used to diagnose diabetes (26). If the patient’s diastolic blood pressure was more than 90 mmHg, their systolic blood pressure was more than 140 mmHg, or they were taking blood pressure medicine as prescribed by a medical expert, these conditions were considered hypertension.

We chose the factors for our investigation that can skew the association between food intake and health outcomes based on the theoretical framework and available literature on the effect of exercise on the relationship between dietary fiber and constipation (15, 22). These variables include demographics (age, gender, race/ethnicity), socioeconomic status (income, education), lifestyle factors (physical activity, smoking status), and clinical features (body mass index, current health status). By including these variables, we want to take into consideration any confounding variables that might affect the findings of our study and make sure that the correlations we find are more likely to be representative of real dietary practices.

### 2.4 Statistical analysis

Empower Stats (version 2.0) and R software (version 4.1.3) were used to conduct descriptive analyses for each participant, with a significance level of *p* < 0.05. Individuals who had missing covariate data were not included in the study. Three groups were created based on dietary consumption of vitamin B6, with the first category (T1) serving as the standard. Whereas categorical data were reported in percentages, continuous data were evaluated using the mean, standard deviation (SD), or median interquartile range (IQR), as applicable. Whereas t-tests were used for continuous data, chi-square tests were used to analyze categorical variables. Using multivariate logistic regression analysis, the association between vitamin B6 consumption and persistent constipation was investigated. Model 2 contained demographic variables such age, gender, and race (27), whereas Model 3 added additional covariates like BMI, marital status, alcohol consumption, smoking behaviors, PIR, hypertension, diabetes, and heart disease. Model 1 was unadjusted. Subgroup analyses based on various factors were also carried out. Restricted cubic spline (RCS) curve fitting was used to provide further insight into the inverse link between vitamin B6 consumption and persistent constipation. These statistical methods made it easier to conduct a comprehensive analysis of any potential links between dietary vitamin B6 consumption and constipation risk.

## 3 Result

### 3.1 Population baseline table

Table 1 summarizes the study’s participants, including 3,643 people who met the screening criteria. 50.04% of these participants identified as male, 49.96% as female, with an average age of 49.39 years (standard deviation of 17.76). Of the total participants, 270 reported constipation, yielding a 7.41% prevalence rate. The subjects were classified into three categories based on their vitamin B6 consumption. In this study, 7.41% of individuals reported constipation, with prevalence rates increasing throughout the tertiles of vitamin B6 intake. The prevalence of constipation was found at 9.23% in tertile 1 (T1), 8.15% in tertile 2 (T2), and 4.85% in tertile 3. Within the three tertiles of vitamin B6 consumption, factors such as age, gender, PIR, race, BMI, diabetes, hypertension, smoking behaviors, and alcohol usage were shown to be statistically significant (all *p* < 0.05).

**Table 1:** Baseline characteristics of the study population according to the vitamin B6 intake in NHANES 2009–2010.

Characteristics	T1 (*n* = 1,213)	T2 (*n* = 1,214)	T3 (*n* = 1,216)	*p*-value	*p*-value^*^
Vitamin B6 intake	1.07 ± 0.27	1.79 ± 0.21	3.28 ± 1.66	<0.001	<0.001
Constipation				<0.001	—
No	1,101 (90.77%)	1,115 (91.85%)	1,157 (95.15%)		
Yes	112 (9.23%)	99 (8.15%)	59 (4.85%)		
Age	50.46 ± 18.29	49.69 ± 17.28	48.02 ± 17.62	0.002	0.002
PIR	2.27 ± 1.51	2.60 ± 1.58	2.63 ± 1.58	<0.001	<0.001
BMI (kg/m^2^)	29.48 ± 6.95	29.49 ± 6.96	28.52 ± 6.27	<0.001	0.001
Gender				<0.001	—
Male	806 (66.45%)	632 (52.06%)	382 (31.41%)		
Female	407 (33.55%)	582 (47.94%)	834 (68.59%)		
Race				0.034	—
Mexican American	241 (19.87%)	217 (17.87%)	218 (17.93%)		
Other Hispanic	134 (11.05%)	111 (9.14%)	114 (9.38%)		
Non-Hispanic White	554 (45.67%)	605 (49.84%)	643 (52.88%)		
Non-Hispanic Black	215 (17.72%)	222 (18.29%)	192 (15.79%)		
Other race—including multi-racial	69 (5.69%)	59 (4.86%)	49 (4.03%)		
Marital status				<0.001	—
Married	582 (47.98%)	661 (54.45%)	646 (53.12%)		
Widowed	128 (10.55%)	109 (8.98%)	70 (5.76%)		
Divorced	160 (13.19%)	126 (10.38%)	126 (10.36%)		
Separated	43 (3.54%)	36 (2.97%)	34 (2.80%)		
Never married	204 (16.82%)	195 (16.06%)	236 (19.41%)		
Living with partner	96 (7.91%)	87 (7.17%)	103 (8.47%)		
Refused	0 (0.00%)	0 (0.00%)	1 (0.08%)		
Alcohol consumption				<0.001	—
No	412 (33.97%)	316 (26.03%)	215 (17.68%)		
Yes	801 (66.03%)	898 (73.97%)	1,001 (82.32%)		
Hypertension				0.003	—
No	744 (61.34%)	768 (63.26%)	824 (67.76%)		
Yes	469 (38.66%)	446 (36.74%)	392 (32.24%)		
Diabetes				<0.001	—
No	1,022 (84.25%)	1,086 (89.46%)	1,105 (90.87%)		
Yes	191 (15.75%)	128 (10.54%)	111 (9.13%)		
Smoking status				0.009	—
No	661 (54.49%)	691 (56.92%)	617 (50.74%)		
Yes	552 (45.51%)	523 (43.08%)	599 (49.26%)		

BMI, body mass index; PIR, the ratio of family income to poverty.

### 3.2 The relationship between vitamin B6 intake and constipation

A multivariate logistic regression analysis was performed (refer to Table 2). We made adjustments for gender, age, race, and marital status in Model 2, and for all possible confounding variables in Model 3. In Model 1, we made no modifications. Higher vitamin B6 levels were linked to a decreased incidence of constipation, according to the weighted logistic regression analysis. Without controlling for any confounders, Model 1’s analysis of the continuous logarithmic form of vitamin B6 showed a significant inverse relationship between vitamin B6 consumption and constipation risk (OR = 0.61, 95% CI = 0.48–0.77, *p* < 0.0001). With an OR of 0.72 (95% CI = 0.56–0.92, *p* = 0.0089), this connection persisted in Model 2 even after controlling for demographic and socioeconomic characteristics. With an OR of 0.72 (95% CI = 0.56–0.93, *p* = 0.0122), the negative association between vitamin B6 and constipation persisted in Model 3 even after further controlling for all possible confounding variables. Higher vitamin B6 intake was associated with a 28% reduction in constipation risk, the relationship between vitamin B6 consumption and constipation risk is independent, indicating that the risk of constipation drops by 28% for every unit increase in the logarithm of vitamin B6 intake, from these researches. This offers a measurable indicator of the beneficial impact of increased vitamin B6 consumption.

**Table 2:** Odds ratios and 95% confidence intervals for constipation according to dietary vitamin B6 intake.

	OR (95% CI), *p*-value		
Continuous	Model 1	Model 2	Model 3
	0.61 (0.48, 0.77) <0.0001	0.72 (0.56, 0.92) 0.0089	0.72 (0.56, 0.93) 0.0122
Categories			
Tertile1	1.0	1.0	1.0
Tertile2	0.87 (0.66, 1.16) 0.3460	0.96 (0.72, 1.27) 0.7541	0.99 (0.74, 1.32) 0.9214
Tertile3	0.50 (0.36, 0.69) <0.0001	0.62 (0.44, 0.87) 0.0054	0.62 (0.44, 0.88) 0.0068

In the sensitivity analysis, vitamin B6 was transformed from a continuous variable into a categorical one (tertiles).

OR stands for odds ratio, while 95% CI refers to the 95% confidence interval.

Model 1: No adjustments were made for covariates.

Model 2: Adjustments were made for sex, age, race, and marital status.

Model 3: Adjusted for sex, age, race, PIR, BMI, smoking habits, alcohol intake, hypertension, diabetes, heart conditions, liver issues, and marital status.

We also transformed the continuous variable of vitamin B6 consumption into categorical variables (split into three quartiles) in order to do a sensitivity analysis. In the model that was fully adjusted, the incidence of constipation was considerably lower for those with the highest consumption of vitamin B6 than for those with the lowest intake (OR = 0.62; 95% CI = 0.44–0.88, *p* = 0.0068). Additionally, those who consumed the most vitamin B6 also experienced less constipation than those who consumed the least, but this difference was not statistically significant (OR = 0.99; 95% CI = 0.74–1.32, *p* = 0.9214).

### 3.3 Subgroup analysis

**Figure 2:**
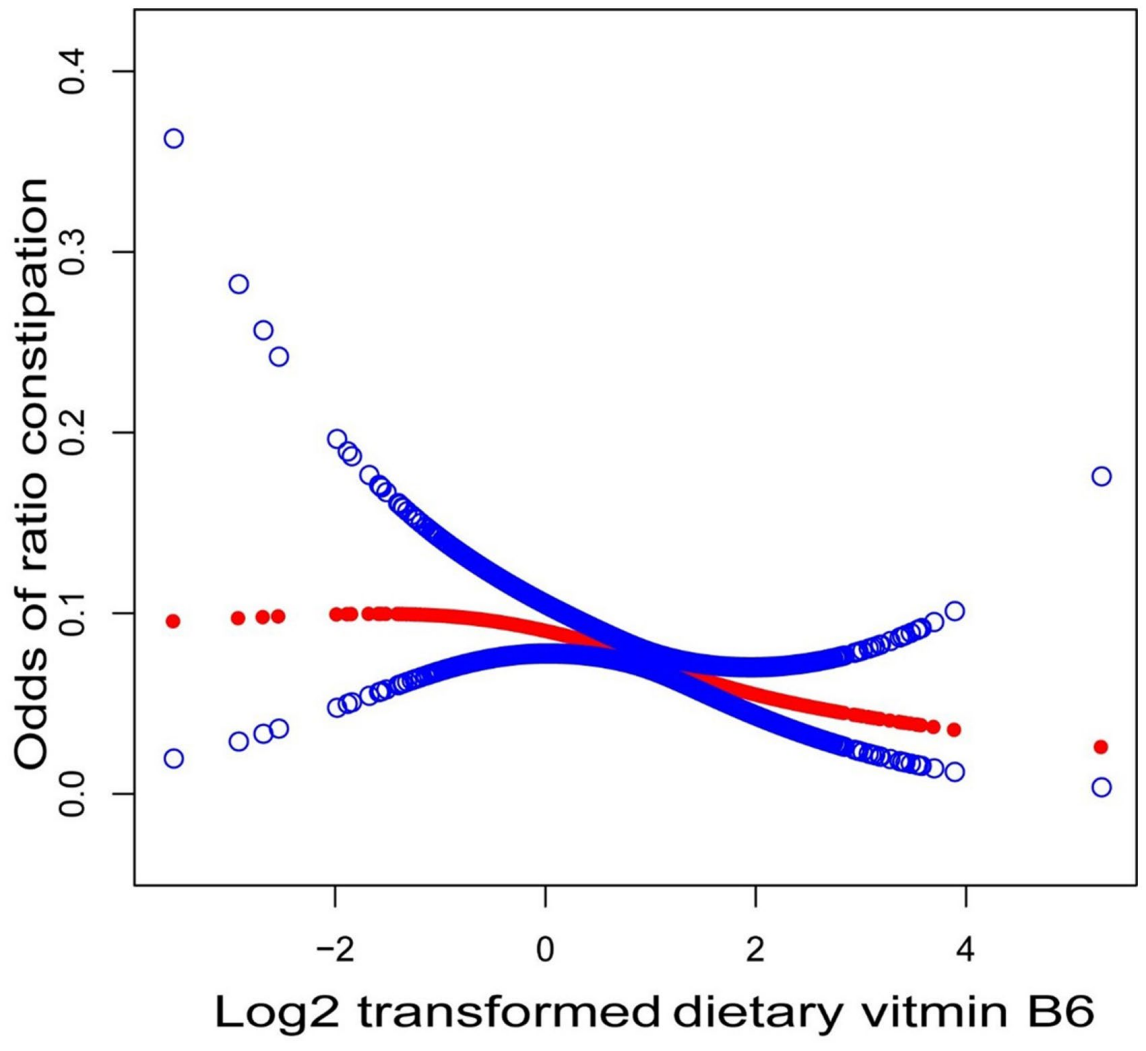
Association between vitamin B6 intake and constipation. In the RCS model, gender, age, race, PIR, marital status, BMI, smoking, drinking, hypertension, diabetes, and heart disease were adjusted.

There was no consistent correlation between vitamin B6 consumption and a reduction in constipation, according to the results of our subgroup analyses, which are shown in Table 3 and Figure 2. An inverse relationship between vitamin B6 consumption and constipation was seen in the female subgroup (OR = 0.54; 95% CI, 0.36–0.81, *p* = 0.0029). Throughout their life cycles, including menstruation, pregnancy, and menopause, women undergo particular hormonal changes. These hormonal shifts can affect how nutrients are metabolized and used, especially B vitamins. The production of neurotransmitters and hormones, which may be more prominent in women because of these hormonal changes, is known to be influenced by vitamin B6. Estrogen is also thought to improve the metabolism of B vitamins, especially B6. The increased protective effect shown in women may be explained by this interaction (28). Furthermore, a negative connection was discovered in the subgroup of Hispanic Black people (OR = 0.67; 95% CI, 0.46–0.97, *p* = 0.0362). Patients with positive hypertension showed statistically significant findings (OR = 0.69; 95% CI, 0.51–0.93, *p* = 0.0156). Additionally, a significant correlation (*p* < 0.05) was discovered between vitamin B6 intake and constipation in those with diabetes. Patients who smoke showed a negative association within the smoking status subgroup. However, the statistical significance of this connection (OR = 0.83; 95% CI, 0.68–1.01, *p* = 0.0664) was not reached. The interaction test’s findings indicated that there were no significant variations in the correlation between vitamin B6 intake and constipation across the various groups. There were no discernible relationships between this negative correlation and any of the following variables: gender, race, smoking behaviors, diabetes, hypertension, or diabetes (all interactions had *p* > 0.05). However, among those who consumed alcohol, a significant negative connection was seen (interaction *p* = 0.043).

**Table 3:** Subgroup analysis of the association between dietary vitamin B6 intake and constipation.

Variable	OR (95% CI)	*p*-value	*p* for interaction
Gender			0.0625
Male	0.88 (0.64, 1.21)	0.4192	
Female	0.54 (0.36, 0.81)	0.0029	
Race			0.2464
Mexican American	0.69 (0.47, 1.01)	0.0547	
Other Hispanic	0.93 (0.66, 1.32)	0.6849	
Non-Hispanic White	0.93 (0.78, 1.12)	0.4547	
Non-Hispanic Black	0.67 (0.46, 0.97)	0.0362	
Mexican American	1.17 (0.70, 1.96)	0.5573	
Hypertension			0.0901
Yes	0.69 (0.51, 0.93)	0.0156	
No	0.91 (0.79, 1.06)	0.226	
Diabetes			0.0543
Yes	0.50 (0.27, 0.93)	0.0292	
No	0.88 (0.77, 1.01)	0.0782	
Smoking status			0.6837
Yes	0.83 (0.68, 1.01)	0.0664	
No	0.88 (0.73, 1.05)	0.1499	
Alcohol consumption			0.043
Yes	0.61 (0.45, 0.83)	0.0016	
No	1.07 (0.68, 1.68)	0.7806	

Analyses of subgroups that incorporate covariates adjusted for in Model 3.

GED, general equivalency diploma; OR, odds ratio; 95% CI, 95% confidence interval.

Drinking alcohol can cause problems with metabolism and food absorption. It is well recognized that alcohol can cause deficits by interfering with the absorption of various B vitamins, particularly vitamin B6. Consequently, boosting vitamin B6 intake may help lessen some of the harmful impacts of alcohol on health by offering protection against problems including liver disease, certain neurological disorders, and constipation that are made worse by alcohol use (29, 30).

The nutritional status of various communities might range greatly. For example, because of dietary variations or micronutrient requirements, women may react differently to vitamin B6 than males. It’s also possible for different ethnic groups to have different eating habits. Changes in dietary practices and general nutritional intake may also have an impact on drinkers’ vitamin B6 status. Consequently, the observed protective benefits may be due to the interactions between vitamin B6 and other common dietary components in these populations.

### 3.4 Non-linear relationship between dietary vitamin B6 intake and constipation

Curve-fitting analysis was used to investigate the negative relationship between vitamin B6 intake and constipation, resulting in the creation of a curve. Figure 2 shows the observed negative linear connection between the incidence of constipation and vitamin B6 consumption.

## 4 Discussion

Using a cross-sectional analysis with 3,643 participants, our study found that those who consumed more vitamin B6 had a lower risk of constipation. The results of the subgroup analysis and interaction tests showed that this link held true for different populations. Our findings suggest that a higher consumption of vitamin B6 acts as a stand-alone preventive measure against constipation. Moreover, our multivariate logistic regression analysis—which took into account relevant confounders—showed that a higher dietary intake of vitamin B6 might help alleviate chronic constipation. Four different types of constipation are identified by the Rome IV criteria: opioid-induced constipation, functional defecation problem, functional constipation, and irritable bowel syndrome (5), which are accepted as typical diagnostic protocols. Previous research has indicated variations in the frequency of constipation based on the type of study and the standards used to assess prevalence (31). For instance, Zhang et al. (32) reported constipation rates of 3.08% based on frequency of defecation and 7.01% based on stool consistency. The Bristol Fecal Form Scale from the Intestinal Health Questionnaire was used in this study to characterize constipation in terms of frequency of bowel movements and texture of the stool. Constipation prevalence was found to be 7.41% in the data, which is consistent with what Liu et al. (33) reported. Scholars have gradually brought attention to potential links between dietary components and long-term constipation through thorough analyses of the NHANES database (34). The need of assessing chronic constipation was highlighted by Liu’s et al. (33) demonstration of a negative connection between dietary phosphorus intake and constipation. Similarly, Yang et al. (35) found that an adequate intake of calories may reduce the probability of constipation in both sexes, indicating a negative correlation between dietary energy intake and constipation. Furthermore, Wang et al. (36) looked at the connection between adult Americans’ dietary carotenoids consumption and chronic constipation. According to their findings, males may benefit from consuming more lycopene, whereas women may be less likely to experience chronic constipation if they consume more alpha carotene. There are six different forms of vitamin B-6 in animals. It is a water-soluble vitamin that was first found to have therapeutic effects in rats. The monophosphorylated derivatives of pyridoxine (PN), pyridoxal (PL), and pyridoxamine (PM) as well as their natural forms are PNP, PLP, and PMP. Pyridoxamine phosphate oxidase (PNPO) and kinase work in tandem to convert PN, PL, and PM to pyridoxal 5′-phosphate (PLP). PLP is the biologically active form of vitamin B-6, participating in more than 150 enzyme-catalyzed processes as an enzyme modulator or cofactor (37).

Vitamin B6, or pyridoxine, is essential for several physiological functions. It is a cofactor in more than 100 enzyme processes, including those that produce neurotransmitters including dopamine, serotonin, and gamma aminobutyric acid (38). Apart from controlling the neurobiological processes linked to mood disorders like anxiety and depression, vitamin B6 may also have additional stress-reduction benefits, such as lowering blood pressure, and may lessen the physiological effects of corticosteroid release (39), which may have an impact on intestinal motility.

There are several facets to the possible relationship between vitamin B6 and constipation that merit more research. First off, vitamin B6’s anti-inflammatory qualities could contribute to the preservation of the intestinal mucosa, which might enhance intestinal health and function in general (40). Second, it has been demonstrated that vitamin B6 controls gut microbiota and inhibits gastrointestinal inflammation, which may have an impact on intestinal motility and stool consistency (41). Furthermore, in lactose intolerant individuals with chronic functional gastrointestinal symptoms, vitamin B6 has been found to be essential for reducing symptoms and gut microbial dysbiosis. This implies that it may have an impact on passage difficulties and stool softness (42).

The results of the study showed that a 6-month course of nutritional supplements comprising vitamins B1, B2, and B6 dramatically decreased bloating, constipation, and abdominal pain—all common symptoms of irritable bowel syndrome (43). A balanced diet that provides an adequate amount of vitamin B6 is good for the digestive system and may help avoid constipation. In conclusion, vitamin B6 consumption is important for digestive health and has a wide range of potential therapeutic uses (44). Additionally, our subgroup analysis showed that individuals with hypertension had a lower risk of constipation for every log2 increase in vitamin B6 intake than individuals without hypertension (hypertension-positive: OR = 0.69; 95% CI, 0.51–0.93; *p* = 0.0156, statistically significant; non-hypertensive patients: OR = 0.91; 95% CI, 0.79–1.06; *p* = 0.226, not statistically significant). This implies that people with hypertension should receive more care. Our results show that increased vitamin B6 intake is independently related with a lower risk of constipation, which is consistent with other research. This suggests that vitamin B6 intake may have a considerable negative influence on constipation in individuals with hypertension.

Nonetheless, it is important to recognize a number of significant limitations of this research. First off, there is no way to draw any conclusions about a causal association between vitamin B6 consumption and constipation because this study is cross-sectional (45). Despite finding a link, we are unable to ascertain whether constipation decreases with increased vitamin B6 intake or whether those who have less constipation typically consume larger amounts of vitamin B6. Cross-sectional studies have this intrinsic restriction, highlighting the need for longitudinal research to determine any possible causal correlations (46). Furthermore, the subjects’ typical vitamin B6 consumption could not be fully captured by our reliance on food recalls. Although self-report and recall were used to gather the data on eating habits and bowel movements, 24-h food diaries might not be a reliable indicator of long-term dietary trends (47–49). Our dependence on food memory may underrepresent the amount of vitamin B6 that people typically consume. Despite its convenience, this method is frequently used in large-scale nutrition research, although it has trouble recording long-term eating patterns (50). The consumption amount of vitamin B6 may be incorrectly categorized due to variations in daily diet and potential memory bias. This restriction might weaken the observed relationships and make our estimations less accurate. Future studies may be able to more closely represent habitual consumption of vitamin B6 with the use of more thorough dietary assessment methods, such as meal frequency questionnaires or long-term repeated 24-h recall. The precision and dependability of the food consumption data included in our study are given top priority by this approach (51, 52). There are still more elements that influence constipation that have not been covered, even when several confounding variables are taken into account (53). To learn more about the connection between dietary vitamin B6 and constipation, longitudinal research is required in the future.

Thirdly, self-reported dietary intake and bowel movements may also lead to under- or over-reporting, impacting data accuracy (17, 54, 55). The actual relationship between vitamin B6 consumption and constipation may be underestimated as a result of this measuring mistake. Lastly, because of variations in metabolism, vitamin B6 consumption, and genetic background. It’s possible that the findings of American study cannot be applied to other populations. This highlights the need to carry out comparable research in various populations and restricts the external validity of our findings. Notwithstanding these drawbacks, our research offers insightful initial information on the possible connection between vitamin B6 consumption and constipation. Future studies should prioritize longitudinal study designs to demonstrate causality, use more thorough nutritional assessment techniques to precisely measure long-term vitamin B6 consumption, and examine this association across other groups in order to overcome these constraints.

## 5 Conclusion

Our research shows a negative correlation between the prevalence of chronic constipation in the adult population as a whole with dietary vitamin B6 consumption. According to this research, consuming more vitamin B6 through food may help to enhance intestinal motility and soften stool, which may help reduce constipation symptoms. Therefore, given the potential of vitamin B6 as a non-pharmacological intervention for constipation, healthcare providers may consider dietary recommendations for patients with chronic constipation, pending further clinical trials. To corroborate our findings, however, further extensive prospective investigations are required.
